# Factors Contributing to Screen Exposure in Preschool Children and Its Associated Outcomes: A Systematic Review

**DOI:** 10.1111/cch.70241

**Published:** 2026-02-03

**Authors:** Maria Inês Gomes, Helena Sousa, Marisa Lousada, Daniela Figueiredo

**Affiliations:** ^1^ RISE‐Health, School of Health Sciences University of Aveiro Aveiro Portugal

**Keywords:** child development, preschool, protective factors, risk factors, screen exposure

## Abstract

**Objectives:**

This systematic review aims to synthesise the current evidence on risk and protective factors that lead to prolonged screen exposure, and to identify both adverse and positive developmental outcomes associated with that screen exposure in preschool children.

**Materials and Methods:**

Following PRISMA 2020 guidelines, a systematic search was conducted across PubMed, Medline, Scopus and Web of Science, including observational studies published from 2017 onwards. A rigorous screening and quality appraisal process was applied using the Joanna Briggs Institute's critical appraisal checklists, resulting in the inclusion of 124 studies.

**Results:**

Risk factors were predominantly parent‐related (e.g., excessive parental screen use, technoference, absence of screen rules), followed by contextual (e.g., low SES, lower parental education) and child‐level factors (e.g., early and unsupervised access to screens). Screen exposure was linked to multiple adverse outcomes across developmental, psychosocial, physical, educational and relational domains (e.g., language delays, cognitive deficits, emotional dysregulation, increased obesity risk and weakened parent–child interactions). Conversely, a smaller subset of studies highlighted protective factors and potential developmental benefits under specific conditions.

**Conclusion:**

Screen exposure in early childhood is a multifaceted phenomenon shaped by individual, familial and contextual determinants. While excessive and unregulated use poses clear developmental risks, structured and intentional media engagement may support developmental benefits. Future longitudinal and experimental research is needed to clarify causal pathways and inform evidence‐based policy and parenting practices.

## Abstract (highlights)

**Summary:**

**What's Known:**

- Excessive screen exposure in preschool children is associated with multiple adverse developmental outcomes.

**What's New:**

- Synthesized evidence from 124 observational studies (2017–2025), offering a comprehensive update post‐AAP 2016 guidelines.
- It identifies a wide range of risk factors: parental, contextual and child‐level risk factors.
- Reports an extensive and diverse set of adverse outcomes across multiple developmental domains: language and cognitive development, psychosocial functioning, physical health and relational impacts.

**Clinical Implications:**

- Parental guidance and support are critical: Clinicians should educate parents on screen exposure limits, content quality and co‐use strategies.
- Interventions should not solely focus on screen exposure quantity, but also qualitative aspects: what, how and with whom screens are used.

## 1 Introduction

In recent decades, the integration of digital devices into daily life has dramatically changed the environments in which children grow and develop (Vedechkina and Borgonovi 2021). Preschool children, typically aged between 2 and 5 years old, are one of the most vulnerable age groups since they are experiencing a rapid period of brain development and forming foundational habits and behaviours that can significantly influence their lifelong health and well‐being (Brown and Jernigan 2012). The prevalence of screen exposure among preschool children has surged, due to the constant presence of screens in households and educational organisations (Yalçin et al. 2021). Nowadays, children spend more time using these digital devices than doing any other leisure activity or playing (Wang et al. 2021; Hinkley et al. 2018).

International guidelines highlight early childhood as a critical period for the establishment of healthy movement and media‐related behaviours. The World Health Organization (WHO) recommends that children aged 3–4 years should accumulate at least 180 min of physical activity per day, should not be restrained or remain sedentary for more than 1 h of sedentary screen time and should obtain 10–13 h of good quality sleep (World Health Organization 2019). These recommendations provide a global public health framework for promoting healthy movement, sedentary behaviour and sleep patterns in early childhood. Complementing this global guidance, the American Academy of Pediatrics (AAP) recommends limiting screen time for children aged 2 to 5 years to no more than 1 h per day of high‐quality programming, emphasising the importance of maintaining a balance between screen‐based activities and other developmental activities, such as physical play, social interaction and sleep (Council on Communications and Media 2016). However, adherence to these guidelines remains challenging for many families, with studies indicating that a significant proportion of preschool children exceed the recommended screen time limits (Yalçin et al. 2021; Gomes et al. 2024).

The rapid evolution of screen technologies presents unique challenges for understanding the full scope of effects associated with screen exposure in preschool children. Moreover, individual differences in screen use patterns, content consumption and environmental contexts further complicate the characterisation of the relationship between screen exposure and child development outcomes (McArthur, Browne, et al. 2022a).

Understanding modifiable risk factors, such as screen content, duration of use and engagement patterns, is critical to mitigating harm and promoting healthy development in childhood. Parents, caregivers and educators should be able to establish informed and balanced practices to support physical, psychological, cognitive and socio‐emotional well‐being. Moreover, recognising protective factors, such as parental mediation strategies, co‐viewing experiences and screen‐free alternatives, can enhance resilience and protect against the negative consequences of screen exposure (Morgan et al. 2021).

Despite several high‐quality systematic reviews examining the associations between health indicators and physical activity (Carson, Lee, et al. 2017), combinations of movement (Kuzik et al. 2017), sleep (Chaput et al. 2017) and sedentary behaviour (including screen time) (Poitras et al. 2017) in early childhood, particularly those conducted to inform the Canadian (Tremblay et al. 2017) and the Australian 24‐Hour Movement Guidelines (Okely et al. 2017) published in 2017 (Veldman et al. 2023; Jourdren et al. 2023; Ahmer et al. 2025). Since this period, and particularly following the COVID‐19 pandemic, preschool children's exposure to digital technologies has increased markedly, alongside qualitative shifts in the function and integration of screen use in daily life. Evidence points not only to higher overall screen time, but also to meaningful changes in the contexts and purposes of digital media in children's lives (Hedderson et al. 2023; Fitzpatrick et al. 2022), with recent findings indicating that families play a critical role in how and how much children engage with screens (Gomes et al. 2024). Furthermore, to the authors' knowledge, no recent systematic review has comprehensively gathered the risk and protective factors associated with screen use in preschool‐aged children, nor has it simultaneously systematized the adverse effects and potential benefits across multiple domains of development (language, cognition, socio‐emotional, physical, educational and relational).

The main objective of this systematic review is to identify and synthesize the evidence on risk and/or protective factors for increased screen exposure and/or its adverse and/or beneficial impacts on preschool children. By synthesising the available evidence and critically evaluating the methodological rigour, consistency of findings and risk of bias, this systematic review aims to provide a comprehensive understanding of the relationship between screen‐related variables and child development outcomes. Ultimately, the findings of this review will provide evidence‐based recommendations and strategies for promoting optimal screen use practices and supporting children's development during the critical early years of life.

## 2 Method

This review was conducted following the 2020 Preferred Reporting Items for Systematic Reviews and Meta‐Analysis (PRISMA) statements (Page et al. 2021) and was registered in the international prospective register of systematic reviews (PROSPERO—number removed to preserve anonymity).

### 2.1 Eligibility Criteria

#### 2.1.1 Population and Exposure

Primary studies were included if they involved preschool children who were exposed to screens. All forms of screen exposure were considered, including (but not limited to) television, computers, tablets and smartphones' use. Studies focusing exclusively on children with neurodevelopmental disorders, such as autism spectrum disorder or attention‐deficit/hyperactivity disorder, were excluded from the current review.

In this review, ‘screen exposure’ was defined broadly and multidimensionally, in line with recent advances in the literature, ensuring that the review captures the complexity of preschool children's media experiences beyond screen time alone. Screen exposure was not limited to the duration of use (screen time), but also the type of device (TV, smartphones, tablets, computers), content (education vs. entertainment) and setting (e.g., co‐viewing with caregivers, parental mediation, free access). Eligible studies were included if they reported any of these dimensions of exposure.

#### 2.1.2 Research Outcomes

Studies were considered eligible if they examined risk and/or protective factors for increased screen exposure and/or reported their adverse and/or beneficial impacts on the target population.

#### 2.1.3 Studies' Design

Observational research (cohort, cross‐sectional and case–control studies) published after (and including) 2017 was admitted. The rationale for selecting this time frame is that the AAP published updated guidelines on children's screen media use in November 2016 (Council on Communications and Media 2016). These guidelines had a substantial impact on research practices and parental counselling worldwide and therefore studies published from 2017 are expected to reflect contemporary approaches to screen exposure and its associated outcomes. Other reviews, interventional studies and grey literature, namely study protocols, conference abstracts, book chapters, commentaries and theses were excluded. Articles written in languages other than English, Portuguese or Spanish were also not considered.

### 2.2 Search Strategy

The literature search was conducted in four electronic indexing databases: PubMed, Medline, Scopus and Web of Science. The search was conducted between May 2024 and February 2025 and was restricted to studies published in peer‐reviewed journals. The following keywords were used interchangeably: ‘preschool child*’ OR ‘early child*’ OR preschool AND ‘screen* exposure*’ OR ‘screen* us*’ OR ‘screen time’ OR ‘media us*’ OR ‘media exposure’ OR ‘screen media’ OR ‘digital device*’ OR ‘mobile phone*’ OR ‘smartphone*’ OR ‘tablet*’ OR ‘television*’ OR ‘display*’ AND (‘risk factor*’ OR ‘protective factor*’ OR ‘parent* supervision’ OR ‘socioeconomic factor*’ OR ‘environmental factor*’) OR (‘adverse effect*’ OR ‘positive effect*’ OR ‘child development’ OR ‘language development’ OR ‘language acquisition’ OR ‘cognitive development’ OR ‘social–emotional development’ OR ‘physical health outcome*’ OR ‘psychomotor skill*’ OR ‘developmental outcome*’).

### 2.3 Study Selection and Data Extraction

All records were extracted to Mendeley – Reference Management Software, and duplicates were automatically removed. Unique studies were initially labelled as included, excluded or unclear based on the title and abstract, and the full text was subsequently retrieved for those deemed unclear or included. These articles were then screened for eligibility independently by the two reviewers [Author1 and Author2]. Discrepancies were resolved by discussion until a consensus was reached. In turn, data extraction was performed by one author [Author1] and confirmed by a second author [Author2], following the JBI Manual for Evidence Synthesis Chapter 7 ‘Systematic reviews of etiology and risk’ data extraction guidelines (*JBI Manual for Evidence Synthesis* 2024).

### 2.4 Critical Appraisal

Critical appraisal of the included studies was performed using the Joanna Briggs Institute (JBI) Statistics Assessment and Review Instruments checklists for cross‐sectional or cohort studies (Moola et al. 2020). Two authors [Author1 and Author2] independently rated each study; disagreements were resolved by debating the methodological quality of each study until reaching a consensus.

### 2.5 Data Systematisation

The Dimensional Model of Early Experience, tested by Hedderson et al. (2023) was used to guide the qualitative systematisation of the results extracted from each primary study. Conceptually, this model proposes that several contextual factors (e.g., socioeconomic status, family income, parental education, parental occupation), home environment indicators (such as parental mental health status and parental screen use), as well as individual child‐related variables (including participation in enriching activities like reading, singing, cooking or exercising) can exert both positive and negative influences on early development (Hendry et al. 2023). In light of this theoretical approach, through these risk and protective factors, screen exposure can impact children's strengths and difficulties in different cognitive, social and emotional outcomes, including the development of prosocial (predisposition to share voluntarily and being considerate of others' feelings), internalising (tendency to be self‐critical and anxious) and externalising (propensity to ‘act out’ in disruptive or aggressive ways) behaviours (Hendry et al. 2023). Based on this model, the data retrieved from each primary study were categorised by two independent reviewers (Author1 and Author2) and, in case of discrepancies or when there was a need to create new categories to fit the results of the current review, discussion among all authors helped to reach a consensus.

## 3 Results

### 3.1 Included Studies

**FIGURE 1:**
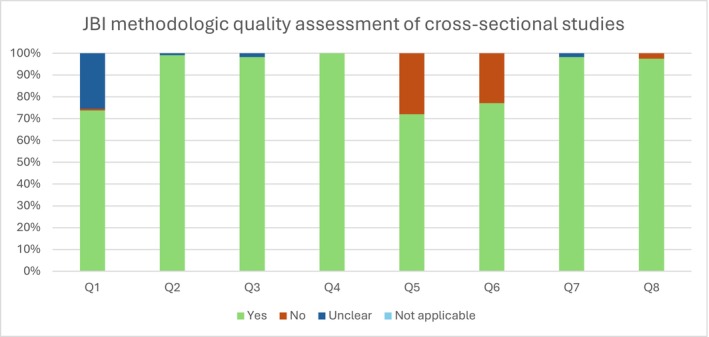
Methodological quality assessment of included cross‐sectional studies using the Joanna Briggs Institute (JBI) Critical Appraisal Checklist for Analytical Cross‐Sectional Studies. Bars represent the percentage of studies rated as ‘Yes’, ‘No’, ‘Unclear’ or ‘Not applicable’ for each checklist item.

Figure 1 presents the PRISMA 2020 flow diagram with the different stages of study selection, including the reasons for the studies' exclusion. A total of 124 studies were included in the current systematic review.

### 3.2 Critical Appraisal Results

**FIGURE 2:**
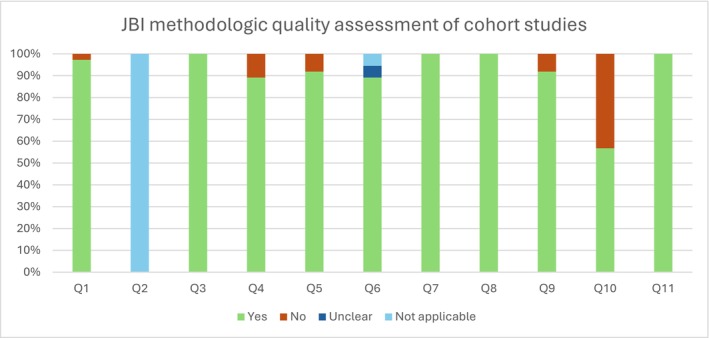
Methodological quality assessment of included cohort studies using the Joanna Briggs Institute (JBI) Critical Appraisal Checklist for Analytical Cross‐Sectional Studies. Bars represent the percentage of studies rated as ‘Yes’, ‘No’, ‘Unclear’ or ‘Not applicable’ for each checklist item.

An inter‐rater agreement of 91.9% was obtained between the two authors (Author1 and Author2) who conducted the critical evaluation of the 205 records assessed for eligibility using the full text. Cross‐sectional studies were assessed using the Checklist for Analytical Cross‐Sectional Studies and cohort studies were assessed using the Checklist for Cohort Studies (Moola et al. 2020), with the results of the critical appraisal summarised graphically in Figures 1 and 2, respectively.

**FIGURE 3:**
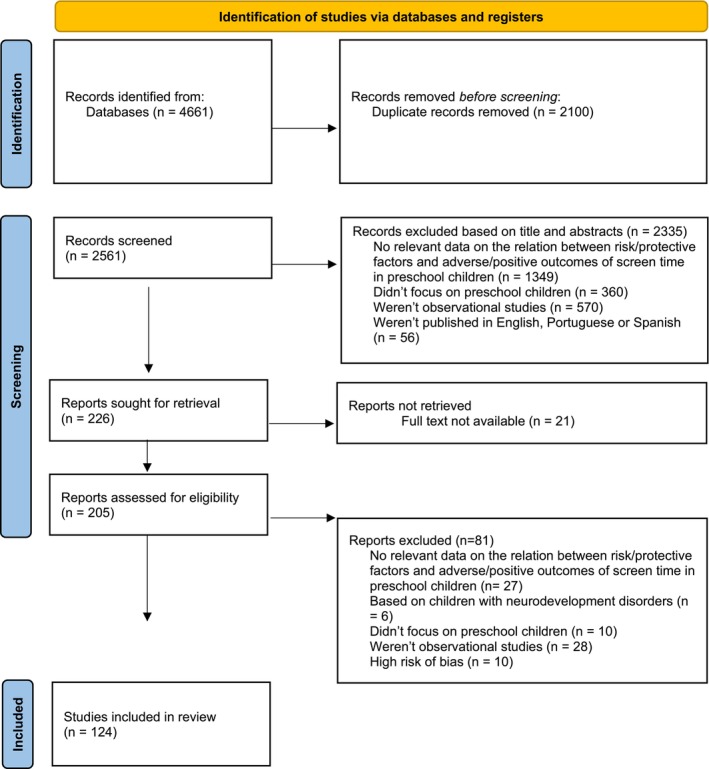
PRISMA flow diagram.

Overall, of the primary studies reviewed, 27% of the cross‐sectional studies did not report complete eligibility criteria (for instance, some omitted whether and how the presence of neurodevelopmental disorders in the target population was considered as an exclusion criterion), while 35.63% failed to identify or address confounding factors. In turn, 98% of the primary studies employed appropriate statistical analysis considering their research objectives, 2% did not clearly describe the methodology used to assess screen exposure and 8% of cohort studies lacked information on follow‐up procedures, including reasons for attrition. In the end, after a careful debate between all authors, 10 studies were of poor methodological quality and were excluded (Figure 3).

### 3.3 Study Characteristics

This review included 124 observational studies, comprising both cross‐sectional (74.2%, *n* = 92) and cohort designs (25.8%, *n* = 32). In general, sample sizes ranged from 36 to 53 575 participants, recruited from both Western (e.g., United States, Australia, Germany) and Eastern cultures (e.g., Singapore, Japan, Hong Kong); gender distribution was balanced, with a slight predominance of female participants. Most of the investigations focused on the adverse effects of increased screen exposure (75%, *n* = 93), followed by its risk factors (31%, *n* = 38). Only 4% (*n* = 5) of the selected studies identified protective variables against prolonged screen exposure, and 8% (*n* = 10) reported their beneficial effects among the target population. Overall, data focused on diverse contextual, child‐ and parent‐related factors and/or the effects of longer screen exposure on cognitive, social–emotional, physical and language outcomes, with a particular emphasis on understanding how screen exposure impacted early childhood development.

### 3.4 Risk Factors That Lead to Screen Exposure

Table 1 presents 86 risk factors that lead to screen exposure in preschool children, reported in 38 studies, divided into three categories: contextual factors, parent‐level factors and child‐level factors, based on the Dimensional Model of Early Experience proposed by Hedderson et al. (2023).

**TABLE 1:** Risk factors leading to screen exposure in preschool children.

Categories	Risk factors	References
Contextual factors (30) Related to the socioeconomic, cultural and family environment in which the child is inserted.	Low socioeconomic status (SES) (6)	(McArthur et al. 2020; Mattsson et al. 2021; Lan et al. 2020; Grummon et al. 2017; Kracht et al. 2019; Griffith and Qiu 2022)
	Higher household income (2)	(Abdulla et al. 2023; Nobre et al. 2021)
	Non‐white ethnicity (e.g., Malay, Latino, Indian, African American) (4)	(Bernard et al. 2017; McArthur et al. 2020; Cerin et al. 2022; Kracht et al. 2019)
	Migration background (1)	(63)
	Living in urban areas (1)	(Xie et al. 2024)
	Lack of a safe neighbourhood (restricting outdoor play) (3)	(Wang et al. 2021; Mattsson et al. 2021; Cerin et al. 2022)
	Younger maternal age (< 40) (Yalçin et al. 2021)	(Bernard et al. 2017; Poncet et al. 2022; Abdulla et al. 2023)
	Higher parental age (1)	(Kattein et al. 2023)
	Lower parental education (high school or less) (4)	(Bernard et al. 2017; Poncet et al. 2022; Grummon et al. 2017; Yalçın et al. 2022)
	Parents being actively working (1)	(Abdulla et al. 2023)
	Single‐parent family (2)	(Poncet et al. 2022; Grummon et al. 2017)
	Being an only‐child (1)	(Xie et al. 2024)
	Household chaos (e.g., confusion, disorganisation, hurriedness) (1)	(Emond et al. 2018)
Parent‐level factors (Li et al. 2024) Directly concerning the behaviour, emotional state, educational practices and routines of parents.	Increased parental screen use (e.g., longer television viewing time and prolonged smartphone use) (16)	(Wang et al. 2021; Gomes et al. 2024; Bernard et al. 2017; Chen et al. 2019; Poncet et al. 2022; Alqaoud et al. 2021; Abdulla et al. 2023; Sun et al. 2024; Lee et al. 2024; McArthur et al. 2020; Mattsson et al. 2021; Schwarzer et al. 2021; Dy et al. 2023; Kattein et al. 2023; Wu and Ye 2023; Wong et al. 2020)
	Parental phubbing (e.g., higher technoference) (6)	(Alqaoud et al. 2021; Wong et al. 2020; Liu et al. 2024; Li et al. 2024; J. Zhang, Cao, and Li 2024; J. Zhang, Liu, et al. 2024)
	Lack of screen time rules (e.g., granting children screen time on demand and using screens as a tool for child rearing) (5)	(Lee et al. 2024; Wu and Ye 2023; John et al. 2021; Abdullah et al. 2022; Coyne et al. 2022)
	Dysfunctional parenting practices (e.g., being too lax, neglectful or overreactive and using harsh criticism) (3)	(Mattsson et al. 2021; Swit et al. 2023; Domoff et al. 2017)
	Low levels of parent–child closeness (1)	(Swit et al. 2023)
	Maternal sedentarism (2)	(Wang et al. 2021; Chen et al. 2019)
	Parental stress (3)	(Kattein et al. 2023; Grummon et al. 2017; Swindle et al. 2018)
	Parental depression (2)	(Heerman et al. 2017; Park et al. 2018)
	Parental emotional exhaustion (1)	(Sun et al. 2024)
	Parental marital conflict (1)	(Sun et al. 2024)
	Parental need to prioritise chores (1)	(Mattsson et al. 2021)
Child‐level factors (Tremblay et al. 2017) Related to the child's behaviour, emotional characteristics or routines.	Easy screen accessibility (e.g., presence of screen devices in the bedroom, using screens during mealtime) (5)	(Wang et al. 2021; Mattsson et al. 2021; Wu and Ye 2023; John et al. 2021; Yalçın et al. 2022)
	Using screen devices other than computers (e.g., using smartphones for games or social media, watching TV shows or videos for entertainment) (3)	(John et al. 2021; Abdullah et al. 2022; Padmapriya et al. 2021)
	Male gender (3)	(Abdullah et al. 2022; Xie et al. 2024; Yalçın et al. 2022)
	Starting device use at a young age (1)	(Abdullah et al. 2022)
	Being alone during screen use (1)	(Dy et al. 2023)
	Non‐compliance with parental screen rules (1)	(Yalçın et al. 2022)
	Negative emotionality (e.g., dysregulation, lability, negativity) (1)	(Domoff et al. 2017)

Within the contextual factors, the most frequently identified risk factors were low socioeconomic status (6; 18.8%), non‐white ethnicity (4; 12.5%) and lower parental education (4; 12.5%). Regarding the parent‐level factors, excessive screen use by parents (16; 38.1%), parental phubbing (6; 14.3%) and the absence of screen time rules (5; 11.9%) were predominant. Lastly, concerning child‐level factors, the most common were easy access to devices (5; 41.7%) and the use of devices for entertainment purposes (3; 25%).

### 3.5 Protective Factors That Prevent Screen Exposure

Table 2 presents 5 protective factors, reported in 10 studies, divided into two categories: parent‐level factors (3; 60%) and child‐level factors (2; 40%), based on the Dimensional Model of Early Experience proposed by Hedderson et al. (2023).

**TABLE 2:** Protective factors that prevent screen exposure in preschool children.

Categories	Protective factors	References
Parent‐level factors (3) Related to the behaviour, emotional state, educational practices and routines of parents.	Parents limiting their screen time while the child is present (1)	(Bernard et al. 2017)
	Parent–child reading time (1)	(Huang et al. 2024)
	Positive parental attitude on screen time (1)	(Mansor et al. 2021)
Child‐level factors (2) Related to the child's behaviour, emotional characteristics or routines.	Children's energetic mood (related to a better transition away from media) (Vedechkina and Borgonovi 2021)	(Coyne et al. 2022)
	Using smartphones for education, games and social networking (Vedechkina and Borgonovi 2021)	(Park and Park 2021)

Within the parent‐level factors, the studies mentioned the limitation of screen time by the parents in the presence of the child (1; 33.3%), shared reading time between parents and children (1; 33.3%) and the parents' positive attitude towards screen time (1; 33.3%) as protective factors against screen exposure in preschool children. Among the child‐level factors, protective factors included an energetic children's mood (1; 50%) and the use of devices for educational or social purposes (1; 50%).

### 3.6 Adverse Outcomes Associated With Screen Exposure

Table 3 identifies 128 adverse outcomes that come from screen exposure in preschool children, reported in 93 studies, divided into six categories: developmental difficulties (52; 40.6%), psychosocial difficulties (38; 29.7%), physical health difficulties (20; 15.6%), lifestyle impacts (10; 7.8%), educational difficulties (4; 3.1%) and parent–child relationships (2; 1.6%), created considering the Dimensional Model of Early Experience proposed by Hedderson et al. (2023).

**TABLE 3:** Adverse outcomes associated with screen exposure in preschool children.

Categories	Adverse outcomes	References
Developmental difficulties (52) Problems in fundamental domains of child cognitive, language and motor development.	Lower global development (5)	(Dy et al. 2023; Madigan et al. 2019; Zheng and Sun 2021; Oflu and Yalçın 2023; Gath et al. 2025)
	Lower language development (poorer vocabulary; receptive vocabulary; expressive skills; difficulty in understanding sentences; worse structural language; worse pragmatic skills) (21)	(Gomes et al. 2024; Schwarzer et al. 2021; Carson, Rahman, and Wiebe 2017; Moon et al. 2019; Schlesinger et al. 2019; Dynia et al. 2021; McArthur, Tough, and Madigan 2022b; Kerai et al. 2022; Mulé et al. 2022; Mustonen et al. 2022; Rithipukdee and Kusol 2022; Z. Zhang et al. 2022; Gath et al. 2023; Valcárcel Jiménez et al. 2023; Lampis et al. 2023; Monteiro et al. 2023; Rayce et al. 2024; Xie et al. 2024; Novikova et al. 2025; Sundqvist et al. 2025; Sundqvist et al. 2024)
	Lower communication (2)	(Kerai et al. 2022; Axelsson et al. 2022)
	Lower cognitive development (7)	(Kerai et al. 2022; Xie et al. 2024; McNeill et al. 2021)
	Lower gross motor skills development (3)	(Kampouri et al. 2017; Martins et al. 2020; Kracht et al. 2020)
	Lower fine motor skills development (1)	(Zheng and Sun 2021)
	Deficits in attention (6)	(John et al. 2021; Axelsson et al. 2022; Hetherington et al. 2020; Almeida et al. 2023; Kiing et al. 2024; Kaur et al. 2024)
	Lower development of executive functions skills (7)	(Radesky et al. 2022)
	Visual–spatial working memory (1)	(McNeill et al. 2021)
Psychosocial difficulties (Kattein et al. 2023) Challenges in a child's emotional, behavioural and social functioning, including externalising and internalising problems and forming and maintaining prosocial behaviours.	Poor emotional self‐regulation (4)	(Xie et al. 2024; Griffith and Qiu 2022; Hetherington et al. 2020; Choe et al. 2023)
	Behavioural dysregulation (2)	(Kiing et al. 2024; Neville et al. 2021)
	Lower social skills (6)	(Hinkley et al. 2018; Schwarzer et al. 2021; John et al. 2021; Kerai et al. 2022)
	Child psychosocial difficulties (e.g., emotional problems, peer relationship problems, conduct problems, anger, frustration) (13)	(Alqaoud et al. 2021; Abdulla et al. 2023; Wong et al. 2020; Lampis et al. 2023; Liu et al. 2021; McNeill et al. 2021; Christian et al. 2022; Radesky et al. 2022; Fitzpatrick et al. 2023; Owais et al. 2024; Wannapaschaiyong et al. 2023; Huang et al. 2025; Al‐Mehmadi et al. 2024)
	Externalising behaviours (e.g., aggression, rule breaking, hyperactivity) (7)	(McArthur et al. 2020; Christian et al. 2022; Carson et al. 2019; Tamana et al. 2019)
	Internalising behaviours (e.g., fearfulness, social withdrawal, somatic complaints) (4)	(McArthur, Browne, et al. 2022a; McArthur et al. 2020; Carson et al. 2019; Tamana et al. 2019)
	Increased risk of autistic‐like behaviours (1)	(Chen et al. 2021)
	Persistent requesting of screens (1)	(Domoff et al. 2021)
Physical health difficulties (Hedderson et al. 2023) Negative effects on the child's body and physical health.	Overweight/obesity (13)	(Alqaoud et al. 2021; Mattsson et al. 2021; Grummon et al. 2017; Padmapriya et al. 2021; Martins et al. 2020; McNeill et al. 2019; Nasreddine et al. 2017; Kurspahić‐Mujčić and Mujčić 2020; Nobre et al. 2022; Özkaya et al. 2022; Reyna‐Vargas et al. 2022; Ye et al. 2023; Frate et al. 2019)
	Worse cardiovascular health (e.g., higher blood pressure) (2)	(Climie et al. 2022; Pedersen et al. 2021)
	Higher degree of fatigue (1)	(Huang and Duan 2023)
	Ocular morbidity (higher risk of myopia, astigmatism, ametropia; lower visual acuity) (4)	(Huang et al. 2021; Ibrahim et al. 2021; Yang et al. 2022; L. Zhang et al. 2023)
Lifestyle impacts (Morgan et al. 2021) Related to daily health behaviours (nutrition, sleep, physical activity).	Lower physical activity (2)	(Staiano et al. 2018; Cerin et al. 2022)
	Lower dietary variety and adequacy (e.g., increased sugar‐sweetened beverage intake) (2)	(Barros et al. 2019; Quick 2018)
	Sleep issues (e.g., shorter sleep duration, poorer sleep quality, sleep habits, teeth grinding) (6)	(Lan et al. 2020; Baukienė et al. 2021; Axelsson et al. 2022; Zerón‐Rugerio et al. 2024; Grummon et al. 2017; Günay Molu et al. 2021)
Educational difficulties (4) Aspects linked to academic performance and learning.	Poorer school performance (e.g., mathematics achievement, science performance) (2)	(Zheng and Sun 2021; Oflu and Yalçın 2023; Gath et al. 2025; Kampouri et al. 2017; Yang et al. 2024; Cliff et al. 2017; Martins et al. 2020; Kracht et al. 2020; Hetherington et al. 2020; Almeida et al. 2023; Kiing et al. 2024; Kaur et al. 2024; Hu et al. 2020)
	Lower curiosity at kindergarten (2)	(Pınar et al. 2021; Shah et al. 2021)
Parent–child relationship difficulties (2) Refers to the emotional connection, responsiveness and communication between parents and children.	Lower parental interactions (1)	(Topothai et al. 2022)
	Poor parent–child closeness (1)	(Gath et al. 2023)

In the developmental difficulties category, language development deficits (21; 40.4%), lower cognitive development (7; 13.5%) and difficulties with executive functions (7; 13.5%) were the main adverse outcomes identified. Among the psychosocial category, child psychosocial difficulties (13; 34.2%), externalising behaviours (7; 18.4%) and low social skills (6; 15.8%) were highlighted. In terms of physical health difficulties, the most prevalent outcomes were excess weight/obesity (13; 65%), eye morbidity (4; 20%) and impaired cardiovascular health (2; 10%). In terms of lifestyle impacts, the main problems were sleep disturbances (6; 60%), followed by reduced physical activity (2; 20%) and low dietary diversity (2; 20%). Lastly, in the educational category, the most mentioned outcome was poor school performance (2; 50%) and in the parent–child relationship category, studies highlighted poor parental interaction (1; 50%) and parent–child closeness (1; 50%).

### 3.7 Positive Outcomes Associated With Screen Exposure

Table 4 presents 10 positive outcomes, reported in five studies, all of which fell into the developmental strengths category, based on the Dimensional Model of Early Experience proposed by Hedderson et al. (2023).

**TABLE 4:** Positive outcomes associated with screen exposure in preschool children.

Categories	Positive outcomes	References
Developmental strengths (10) Gains in fundamental domains of child cognitive, language and motor development.	Language development (e.g., higher phonological working memory, better use of contextual information) (3)	(McNeill et al. 2021) (Nobre et al. 2021) (Lampis et al. 2023)
	Cognitive development (2)	(Ekholuenetale et al. 2020) (O'connor et al. 2020)
	Fine motor skills development (2)	(Moon et al. 2019) (Souto et al. 2020)
	Global development (1)	(Topothai et al. 2024)
	Non‐verbal reasoning (1)	(Yang et al. 2024)
	Gross motor development (1)	(Chaibal and Chaiyakul 2022)

The most recurrent positive outcomes associated with screen exposure in preschool children were language (3; 30%), cognitive (2; 20%) and fine motor skills (2; 20%) development.

## 4 Discussion

The main findings of this systematic review confirm and deepen the current body of research on the causes and effects of prolonged screen exposure on preschool children. These results also highlight that much of the current literature continues to operationalise screen exposure primarily in terms of screen time, with less systematic focus on content, context or device. Nevertheless, several included studies did differentiate between entertainment versus educational content, active versus passive use and parental mediation practices, underscoring the need for more precise conceptualisations in future research. By synthesising more recent evidence published since the reviews underpinning the 24‐h movement guidelines (Carson, Lee, et al. 2017; Kuzik et al. 2017; Chaput et al. 2017; Poitras et al. 2017), this review extends previous work by integrating risk and protective factors alongside both adverse and potential positive outcomes across multiple developmental domains within a single conceptual framework. The results show that extended exposure to screens during early childhood can lead to diverse outcomes. They must be understood in the context of a broader family and socio‐cultural ecosystem and support the need to develop multidimensional and contextually sensitive interventions aimed at various levels of the child's environment—from the behaviour of parents and caregivers to public policies and institutional practices.

Regarding the risk factors, the prevalence of parental factors such as excessive screen use by caregivers (Wang et al. 2021; Gomes et al. 2024; Bernard et al. 2017; Chen et al. 2019; Poncet et al. 2022; Alqaoud et al. 2021; Abdulla et al. 2023; Sun et al. 2024; Lee et al. 2024; McArthur et al. 2020; Mattsson et al. 2021; Schwarzer et al. 2021; Dy et al. 2023; Kattein et al. 2023; Wu and Ye 2023; Wong et al. 2020), phubbing (Alqaoud et al. 2021; Wong et al. 2020; Liu et al. 2024; Li et al. 2024; J. Zhang, Cao, and Li 2024; J. Zhang, Liu, et al. 2024) and the absence of clear and consistent rules around the time and type of media content (Lee et al. 2024; Wu and Ye 2023; John et al. 2021; Abdullah et al. 2022; Coyne et al. 2022) highlights a significant gap in the guidance offered to families. The implementation of clear parental guides, which provide practical recommendations on duration, context of use and quality of content, should be a priority for health professionals, educators and policymakers and can have a significant impact, warning on the less visible effects of parental digital distraction, such as a reduction in emotional responsiveness and the quality of face‐to‐face interactions (Kumar Muppalla et al. 2023), crucial aspects for the child's emotional and linguistic development (Massaroni et al. 2024).

Considering the contextual factors, it is crucial to underline that in families experiencing multiple vulnerabilities, screens can be perceived as the only viable solution to occupy and stimulate children, especially when parents face a heavy workload, lack of a support network, or emotional difficulties (Nomaguchi et al. 2021). Therefore, broader policy responses, such as support for positive parenting, the provision of community resources (e.g., libraries, children's public spaces) and the promotion of safer and more accessible digital environments, are key to reducing children's screen time (Ponti et al. 2017).

The adverse effects associated with screen exposure in childhood are wide‐ranging, affecting multiple domains of development. The most frequently reported area was language development, with 21 studies reporting delays or deficits in vocabulary, verbal comprehension and expressive language use (Gomes et al. 2024; Schwarzer et al. 2021; Carson, Rahman, and Wiebe 2017; Moon et al. 2019; Schlesinger et al. 2019; Dynia et al. 2021; McArthur, Tough, and Madigan 2022b; Kerai et al. 2022; Mulé et al. 2022; Mustonen et al. 2022; Rithipukdee and Kusol 2022; Z. Zhang et al. 2022; Gath et al. 2023; Valcárcel Jiménez et al. 2023; Lampis et al. 2023; Monteiro et al. 2023; Rayce et al. 2024; Xie et al. 2024; Novikova et al. 2025; Sundqvist et al. 2025; Sundqvist et al. 2024). Language development in early childhood is strongly dependent on reciprocal social interactions rich in verbal stimuli, such as dialogue with adults, shared reading and involvement in symbolic play (Creaghe et al. 2021). These interactions not only expose children to a diverse vocabulary but also provide them with pragmatic and contextual models of language use (Kulkarni and Waknis 2024), while prolonged exposure to screens, particularly to non‐interactive content with low linguistic value, tends to reduce the quantity and quality of linguistic input to which children are exposed, while reducing the time available for active language practice, compromising its development (Gath et al. 2023).

The prolonged screen exposure has also been associated with quick stimuli, immediate rewards and little demand for emotional self‐regulation, factors that can compromise the development of fundamental psychosocial skills, such as inhibitory control, frustration tolerance and conflict resolution, as shown in this review in 13 studies (Alqaoud et al. 2021; Abdulla et al. 2023; Wong et al. 2020; Lampis et al. 2023; Liu et al. 2021; McNeill et al. 2021; Christian et al. 2022; Radesky et al. 2022; Fitzpatrick et al. 2023; Owais et al. 2024; Wannapaschaiyong et al. 2023; Huang et al. 2025; Al‐Mehmadi et al. 2024). The time spent in front of screens often replaces meaningful social interactions and moments of symbolic play, which are crucial for learning social norms and developing empathy (Skalická et al. 2019). The reduction in these interpersonal opportunities may partly explain the effects observed in terms of difficulties with relationships with peers and lower social competence (McArthur, Browne, et al. 2022a; McArthur et al. 2020; Carson et al. 2019; Tamana et al. 2019).

Regarding the physical health difficulties and lifestyle impacts, childhood obesity is a widely recognised adverse outcome of early and excessive exposure to screens, considering that screen time often reduces physical activity and promotes sedentary behaviour (Staiano et al. 2018; Cerin et al. 2022), is frequently associated with eating unhealthy foods during use (Barros et al. 2019; Quick 2018) and can impair sleep (Lan et al. 2020; Baukienė et al. 2021; Axelsson et al. 2022; Zerón‐Rugerio et al. 2024; Grummon et al. 2017; Günay Molu et al. 2021). These mechanisms contribute to the creation of an early obesogenic environment, with long‐lasting physical, emotional and social implications.

The diversity of adverse effects identified in this review raises fundamental questions about the role of digital technology in early childhood, especially when integrated unintentionally or negligently into everyday family life. This wide variety of areas affected points to an indirect and cumulative influence, in which prolonged exposure is intertwined with dysfunctional routines, parental stress and family disorganisation.

Despite the prevalence of evidence on risk factors and adverse outcomes, some protective factors and positive results have also been identified, although in smaller quantities. Essentially, studies found that parents limiting their screen time in the presence of the child (Bernard et al. 2017), joint reading time (Huang et al. 2024) and positive attitudes towards digital use (Mansor et al. 2021) emerge as protective factors concerning parental practices. These data are in line with studies that advocate for active and intentional mediation by caregivers as a key moderator of the effects of technology in childhood (Gomes et al. 2024). Additionally, children who present an energetic mood (Coyne et al. 2022) and have active media use (e.g., use smartphones for education, games and social networking) (Park and Park 2021) also tend to cooperate more with the screentime guidelines. Active media use—referring to the intentional and interactive involvement of users with digital content—may promote the development of cognitive and social skills (Gath et al. 2023) when compared to passive media use—implying the mere viewing or consumption of the displayed content without direct interaction—which can limit the development of these skills (Valkenburg et al. 2022).

The few protective factors identified point to a common pattern: the active and relational mediation of screen use, translated into practices such as co‐viewing, consistent boundaries and the integration of digital into contexts of bonding and shared learning. These practices may not eliminate the risks associated with the use of technology, but they can reduce its impact, transforming a potentially disruptive element into a tool for development and connection. It is essential to acknowledge that the designation of variables as ‘risk’ or ‘protective’ factors should not be construed as representing simple dichotomies. For example, although low socioeconomic status or non‐white ethnicity has been associated with increased screen exposure, the current body of evidence does not substantiate the inference that high socioeconomic status or white ethnicity operate as protective factors. Likewise, while a positive parental orientation towards screen use has been identified as protective in certain studies, this does not entail that a negative orientation necessarily constitutes a risk factor. Recognising this nuance is critical to prevent conceptual oversimplification and to underscore that risk and protection should be regarded as analytically distinct constructs rather than as polar opposites.

It should also be noted that, although on a smaller scale, some benefits of using screens in structured contexts have been reported with improvements in language (McNeill et al. 2021), cognitive development (Ekholuenetale et al. 2020) and fine motor skills (Moon et al. 2019). These findings support the idea that the impact of technology is not unidirectional, but depends on the quality of the content, the function of use (entertainment vs. educational) and the surrounding family context. Recent studies indicate that the interactive and shared use of technology can promote emerging skills, as long as it is aligned with parental involvement practices and adapted to the child's developmental level (Chaibal and Chaiyakul 2022; Souto et al. 2020).

The identification of positive effects associated with the use of screens in preschool children raises a central dilemma for the literature on how to recognise the potential of digital devices. The data found in this review suggest that certain uses—intentional, educational, interactive—can promote significant learning, especially in contexts where other resources are scarce. In this sense, the benefits derive not so much from the device itself, but from the relational, pedagogical and affective context in which the technology is inserted.

This evidence reinforces the need to move beyond a dichotomous view that classifies screen exposure as ‘beneficial’ or ‘harmful’ and to adopt a more nuanced perspective that considers the complexity of the factors involved, as well as the active and responsive forms of mediation that make it possible to maximise the benefits and minimise the risks associated with digital exposure in early childhood.

### 4.1 Limitations of the Study

Despite the growing body of literature on screen exposure in preschool children, the results of this review show gaps in research, both in terms of thematic and methodological focus, which limit the advancement of knowledge and the formulation of practical recommendations based on robust evidence.

One of the main limitations identified is the predominance of cross‐sectional observational studies, which makes it difficult to draw causal inferences between risk or protective factors and the outcomes observed. This limitation compromises the understanding of the developmental trajectories associated with early screen use, as well as the identification of critical periods of greater vulnerability or opportunity. The shortage of longitudinal studies represents a priority gap to be filled. Prospective studies, which assess the effects of screen use over time and test concrete interventions (e.g., parental digital literacy programs), are fundamental to understanding the mechanisms underlying the positive and negative effects of digital exposure.

Regarding the results, only a minority of the included studies reported positive outcomes, and in these cases, the data is often based on specific samples or conditions that cannot be generalised (e.g., African or Asian countries). This asymmetry highlights a tendency in the literature to approach the use of screens from a predominantly deficit perspective, disregarding contexts in which technology can function as a tool to promote development, especially when mediated in an intentional and responsive way. In this sense, there is a clear need to investigate the factors that promote positive and enriching use of screens, including identifying effective parenting practices, the most beneficial types of content and digital design features that enhance the child's active involvement. Studies that analyse how different formats (video, game, interactive app), platforms (TV, tablet, smartphone) and purposes of use (education, communication, recreation) interact with the child's level of development and needs are still scarce and should be a priority on research agendas.

Finally, from a conceptual point of view, the literature on exposure to screens is still excessively focused on the amount of time spent using them, devaluing crucial aspects such as the content, the context of interaction and the motivators for use. This quantitative focus, although useful as an initial reference, proves insufficient to fully comprehend the complexity of children's media experience. There is an urgent need to develop more refined assessment tools, capable of distinguishing, for example, between passive and interactive consumption, individual or shared use and specific types of applications or programs.

### 4.2 Implications for Practice and Policy

The findings of this review underscore the importance of targeted interventions to reduce screen exposure and promote healthier media consumption patterns in children. Health professionals should be equipped with up‐to‐date guidelines to counsel parents on setting healthy screen use limits and choosing age‐appropriate, educational content.

In conclusion, while screen exposure is an inevitable part of modern childhood, excessive and unregulated media use poses significant risks to children's cognitive, emotional and physical development. Parental involvement, adherence to screen use guidelines and mindful media consumption are essential in ensuring that technology serves as a tool for healthy development rather than a source of harm. Future research should continue to explore the long‐term effects of screen exposure, with a particular focus on developing strategies to promote healthier screen habits in early childhood.

## Author Contributions

**Maria Inês Gomes:** conceptualization, methodology, data curation, investigation, formal analysis, writing – original draft, writing – review and editing. **Helena Sousa:** methodology, data curation, formal analysis, writing – original draft. **Daniela Figueiredo:** conceptualization, validation, supervision, project administration, writing – review and editing. **Marisa Lousada:** conceptualization, validation, supervision, project administration, writing – review and editing.

## Funding

Maria Inês Gomes has a PhD grant (2023.02614.BD) financed by Fundação para a Ciência e a Tecnologia through FSE (Fundo Social Europeu). This article was supported by National Funds through Fundação para a Ciência e a Tecnologia, I.P., within CINTESIS, R&D Unit (reference UIDP/4255/2020).

## Conflicts of Interest

The authors declare no conflicts of interest.

## Data Sharing

Data sharing not applicable—no new data generated.

## Data Availability

Data sharing not applicable to this article as no datasets were generated or analysed during the current study.
